# Case Report: Successful staged multidisciplinary management of concurrent duodenal papilla adenocarcinoma and cholelithiasis: a rare case in primary hospital

**DOI:** 10.3389/fsurg.2026.1836730

**Published:** 2026-08-31

**Authors:** Quanlei Wang, Xiyin Ye, Shengchang Li, Shun Ruan, Kai Guo, Tiansheng Xie, Suhui Zeng, Gang Wu, Haibo Chen, Denghui You, Lei Zhang, Changqing Xu, Ningjuan Tang, Wanhong Liang, Sangui Wang

**Affiliations:** 1 Department of Surgery, Dongguan Nancheng Hospital, Dongguan, China; 2 Dongguan Institute of Gallbladder Disease Research, Dongguan Nancheng Hospital, Dongguan, China; 3 Department of Pathology, Dongguan Nancheng Hospital, Dongguan, China; 4 Department of Radiology, Dongguan Nancheng Hospital, Dongguan, China; 5 Department of Laboratory, Dongguan Nancheng Hospital, Dongguan, China; 6 Department of Traditional Chinese Medicine Rehabilitation, Dongguan Nancheng Hospital, Dongguan, China; 7 Department of Medical Management, Dongguan Nancheng Hospital, Dongguan, China; 8 Department of Ultrasonography, Dongguan Nancheng Hospital, Dongguan, China; 9 Department of Internal Medicine, Dongguan Nancheng Hospital, Dongguan, China; 10 Department of Nursing, Dongguan Nancheng Hospital, Dongguan, China

**Keywords:** duodenal papillary adenocarcinoma, lymphatic leakage, lymphatic vessel embolization, radical pancreaticoduodenectomy, staged surgery

## Abstract

**Background:**

Duodenal papillary adenocarcinoma is a rare malignant tumor. Its early symptoms are often insidious and frequently coexist with common biliary diseases such as cholelithiasis, leading to missed or delayed diagnosis. Radical pancreaticoduodenectomy is the standard treatment, but postoperative complications like lymphatic leakage and gastroparesis are common and challenging to manage. Currently, clinical reports on the diagnosis and treatment of duodenal papillary adenocarcinoma combined with gallstones in primary care hospitals are relatively scarce.

**Case description:**

This article reports the case of a 71-year-old female patient who presented with upper abdominal pain. Imaging suggested gallstones and common bile duct stones with biliary obstruction, alongside a mass at the duodenal papilla. The patient declined preoperative ERCP biopsy. The treatment team adopted a staged strategy: Stage one involved laparoscopic common bile duct exploration with stone extraction and cholecystectomy, with intraoperative biopsy confirming duodenal papillary adenocarcinoma (moderately to well-differentiated); Stage two consisted of radical pancreaticoduodenectomy, with partial transverse colectomy performed due to suspected tumor infiltration. Postoperatively, the patient developed persistent lymphatic leakage and gastroparesis syndrome. Through multidisciplinary collaboration, a combination of traditional Chinese medicine acupuncture, enhanced nutritional support, anti-infection therapy, and the innovative application of digital subtraction angiography lymphangiography and targeted embolization successfully controlled the lymphatic leakage. After three months of individualized comprehensive treatment in a primary care hospital, the patient was discharged in stable condition.

**Conclusions:**

For patients with duodenal papillary adenocarcinoma combined with acute biliary obstruction, staged surgery is a feasible individualized strategy. The management of complex complications following radical pancreaticoduodenectomy is crucial. It requires a multidisciplinary integrated diagnosis and treatment model in primary care hospitals, combining traditional supportive therapies with modern precision interventional techniques to improve patient outcomes. This case provides a reference for the diagnosis and treatment of such rare and complex situations.

## Introduction

Duodenal papillary adenocarcinoma is a malignant tumor arising from the mucosal glandular epithelium of the duodenal papilla region. Its incidence is extremely low, less than 1 per 100,000, accounting for only about 0.3% to 0.5% of all gastrointestinal malignancies, making it clinically rare (1, 2). The etiology of this disease is not fully understood, and known risk factors include advanced age, duodenal adenoma, and biliary obstruction leading to cholestasis. Due to its insidious and nonspecific early symptoms, often presenting as upper abdominal discomfort, jaundice, or pancreatitis, which overlap with symptoms of common biliary and pancreatic diseases, diagnosis is challenging. Most patients are diagnosed at an advanced stage, resulting in a poor prognosis and high treatment complexity (3).

Clinically, the situation becomes particularly complex when duodenal papillary adenocarcinoma coexists with common biliary diseases such as cholelithiasis. Symptoms of biliary obstruction caused by gallstones may mask or confuse the early manifestations of the tumor, making it prone to missed or delayed diagnosis. Therefore, for patients with obstructive jaundice or biliary dilation, especially elderly patients, it is essential to remain vigilant for the possibility of coexisting duodenal carcinoma during routine diagnosis and treatment for cholelithiasis and to conduct thorough imaging evaluations.

Radical pancreaticoduodenectomy is currently the primary treatment for localized duodenal papillary adenocarcinoma (4). However, this procedure involves extensive resection, significant trauma, and complex anatomical relationships, leading to a high incidence of postoperative complications. Common complications include pancreatic fistula, biliary fistula, delayed gastric emptying (gastroparesis), and lymphatic leakage, which severely impact postoperative recovery and quality of life (5, 6). Therefore, optimizing surgical strategies and effectively preventing and managing postoperative complications are key to improving patient prognosis.

This case report describes a patient diagnosed with early-stage duodenal papillary adenocarcinoma at a primary care hospital after initially presenting with cholelithiasis. As the patient declined invasive preoperative diagnostic procedures, the treatment team innovatively adopted a staged surgical approach: first addressing the acute biliary obstruction and obtaining a pathological diagnosis intraoperatively, followed by radical resection as the second stage after confirming the malignant diagnosis. This strategy balanced the goals of relieving the acute condition, establishing a definitive diagnosis, and achieving curative treatment.

Postoperatively, faced with the challenging complication of persistent lymphatic leakage, the team employed multidisciplinary collaboration, integrating traditional Chinese medicine acupuncture for rehabilitation, modern nutritional support, anti-infection therapy, and ultimately resolving the issue with precise interventional radiology techniques—lymphangiography and embolization. This case not only demonstrates the application value of staged surgery in specific clinical scenarios but also highlights the central role of a multidisciplinary comprehensive treatment model in managing complex perioperative complications. It provides valuable diagnostic and therapeutic insights and experience for handling similar rare and complex clinical situations.

## Case presentation

### Preoperative comprehensive examination

A 71-year-old female patient presented to our outpatient department with a two-month history of episodic dull pain and discomfort in the upper abdomen. Vital signs were as follows: body temperature 36.5 °C; pulse 85 beats per minute; blood pressure 139/79 mmHg; body mass index 25 kg/m^2^. The abdomen was flat, with no percussion tenderness over the liver or kidney areas. Physical examination revealed normal development and bowel sounds.

Laboratory tests showed: total cholesterol (CHO) 5.58 mmol/L, total bilirubin (TBIL) 28.5 µmol/L, direct bilirubin (DBIL) 22.5 µmol/L, gamma-glutamyl transferase (GGT) 600 U/L, alkaline phosphatase (ALP) 194 U/L, total bile acids (TBA) 37.6 µmol/L, alanine aminotransferase (ALT) 101 U/L, aspartate aminotransferase (AST) 97 U/L, and glycated hemoglobin (HbA1C) 6.93%. Tumor markers were mostly within normal limits (alpha-fetoprotein, carcinoembryonic antigen, carbohydrate antigen 125), except for carbohydrate antigen 19-9, which was elevated at 187.95 U/mL.

**Figure 1:**
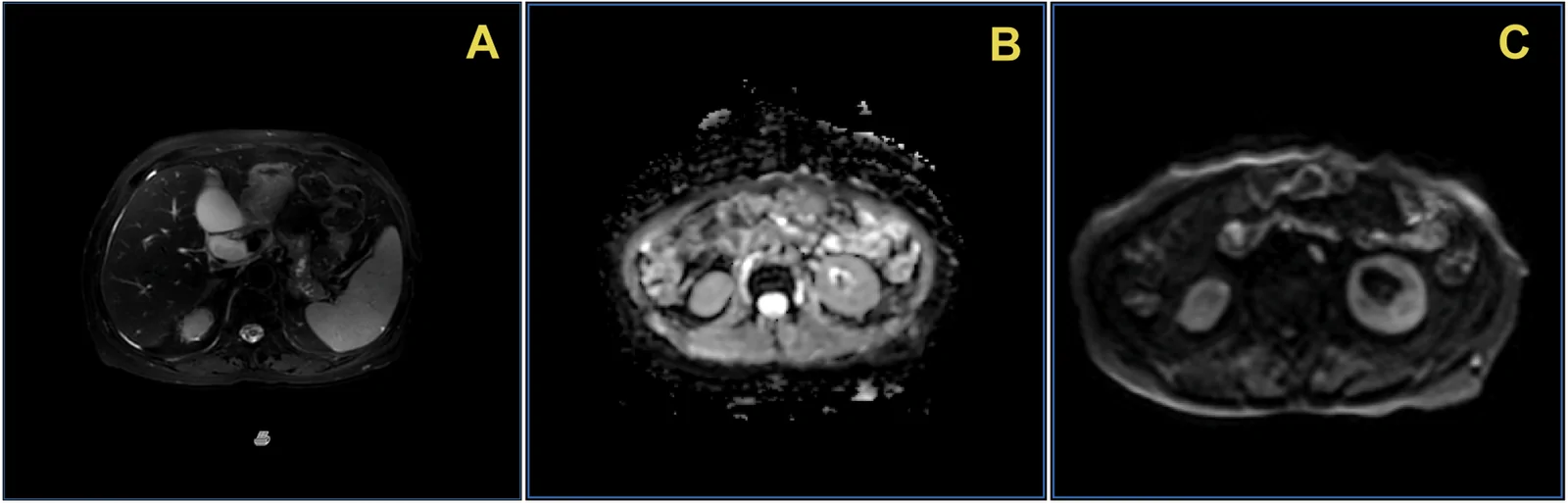
MRI showing gallstones, common bile duct stones, biliary obstruction, and a mass at the duodenal papilla. **(A)** Gallbladder stones and bile duct stones. **(B)** ADC shows low signal (restricted diffusion); and **(C)** DWI shows high signal at the papilla; suggesting mass at the duodenal papilla.

MRI imaging revealed gallbladder enlargement with wall thickening, signs consistent with gallstones and cholecystitis, common bile duct stones, dilation of both the common bile duct and pancreatic duct, and a soft tissue density nodule at the distal end of the common bile duct (duodenal papilla region) (Figure 1). These above findings suggest a diagnosis of gallstones, common bile duct stones, biliary obstruction, and a mass at the duodenal papilla.

### Staged surgical management

Communication with the patient and family regarding test results and potential treatment options took place. The patient declined ERCP and biopsy to further clarify the nature of the duodenal papilla mass. Following multidisciplinary discussion and communication with the patient, laparoscopic common bile duct exploration with stone extraction and cholecystectomy were performed. The surgical procedure was performed under general anesthesia with the patient in the supine position. After establishing pneumoperitoneum and inserting laparoscopic trocars, the gallbladder was retracted. The cystic triangle was dissected to expose the cystic duct and common bile duct; the cystic duct was ligated and divided, and the gallbladder was excised from the liver bed. The hepatoduodenal ligament was then dissected to expose the mid-to-lower segment of the common bile duct, where a longitudinal incision was made. Choledochoscopy revealed an irregular black stone (approximately 1.2 × 0.8 cm) at the distal common bile duct, and a biopsy of the mass at the duodenal papilla was obtained. The procedure was completed uneventfully.

Two weeks after the initial surgery, the patient developed severe nausea and vomiting. Contrast imaging revealed a filling defect at the distal common bile duct with no contrast entering the duodenum. Combined with MRI and biopsy findings, a diagnosis of duodenal papillary adenocarcinoma was established. Given the biliary and digestive obstruction, radical pancreaticoduodenectomy was performed. Surgical procedure: A midline laparotomy was made. After mobilizing the hepatic flexure and performing a Kocher maneuver, the pancreatic head and duodenum were exposed. The stomach was transected approximately 5 cm proximal to the pylorus, and the common bile duct was divided. The gastroduodenal artery was ligated and divided. The pancreas was transected to the left of the superior mesenteric vein, and the pancreatic duct was identified. The pancreatic head and uncinate process were dissected from the portal and superior mesenteric veins. Due to suspected tumor infiltration at adhesion sites between the transverse colon and gastric antrum/duodenum, partial transverse colectomy (8 cm) was performed *en bloc* to ensure R0 resection. The jejunum was divided 15 cm distal to the ligament of Treitz, and the specimen (distal stomach, pancreatic head, duodenum, and proximal jejunum) was removed *en bloc*. Intraoperative frozen section confirmed a moderately to well-differentiated adenocarcinoma with submucosal invasion; no lymphovascular or perineural invasion was identified. Lymphadenectomy of stations 8a, 12a, and 14v was performed. Digestive tract reconstruction: An end-to-side pancreaticojejunostomy (duct-to-mucosa) was performed, followed by an end-to-side choledochojejunostomy 10 cm distally, and an end-to-side gastrojejunostomy 40 cm further distally. Due to tumor adherence, a segmental transverse colectomy (approximately 8 cm) with end-to-side anastomosis was additionally performed. A gastrostomy tube and a jejunal feeding tube were placed. Estimated blood loss was 300 mL. The patient was transferred to the ICU postoperatively.

**Figure 2:**
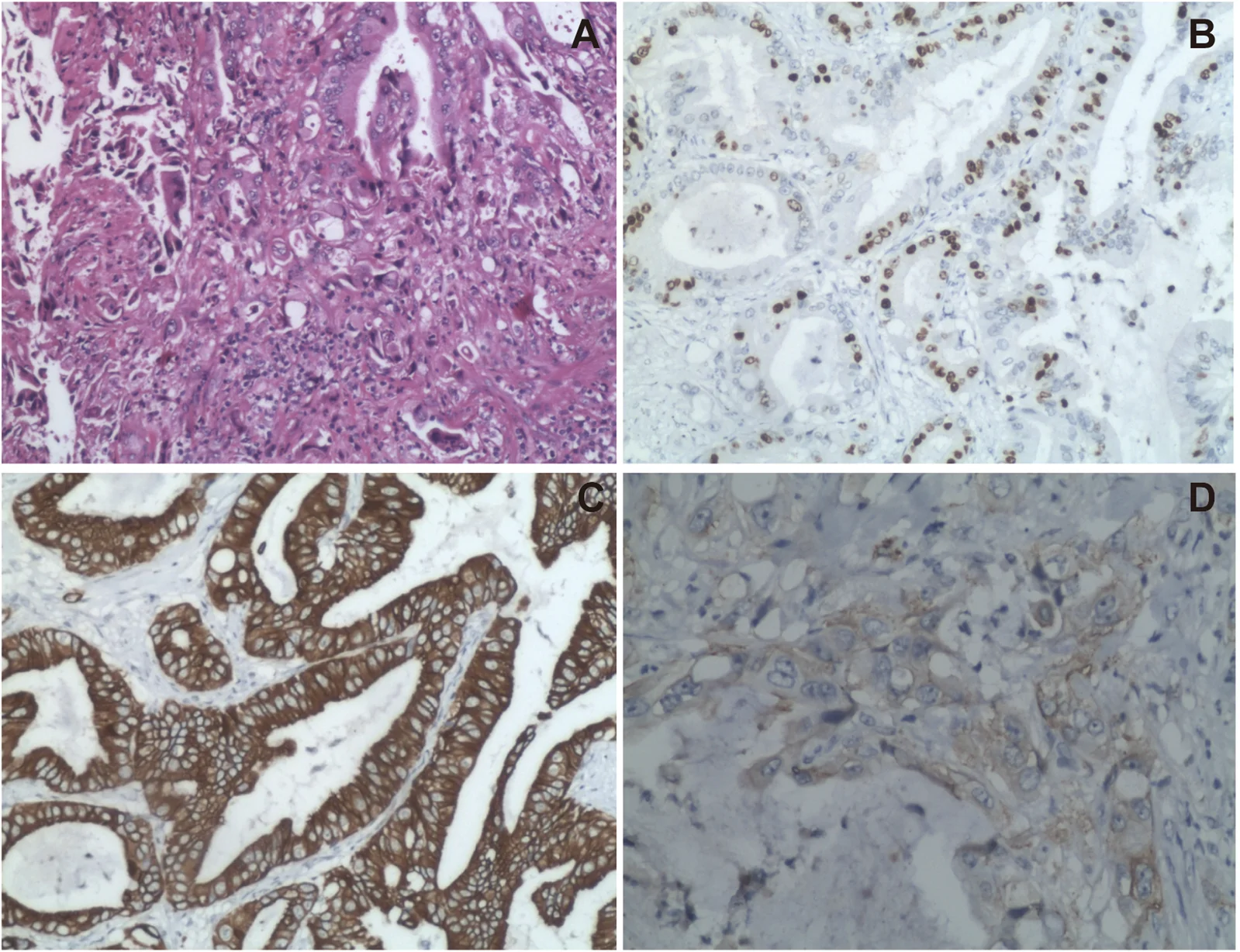
Histopathological and immunohistochemical characteristics of the tumor. **(A)** Hematoxylin and eosin (HE) staining showing invasive growth of the atypical glandular epithelium (×100). Tumor cells demonstrate strong immunoreactivity for **(B)** Ki-67 (nuclear, approximately 40%, ×100), **(C)** CK7 (cytoplasmic, ×100), and **(D)** EGFR(cytoplasmic/membranous, ×100).

Postoperative pathological and immunohistochemical results showed: Duodenal papillary moderately differentiated adenocarcinoma(intestinal-type, based on morphological features showing glandular differentiation). Using the AJCC/UICC 8th edition staging system, the tumor was classified as T1bN1M0, Stage IIIA. Tumor size was 1.5 × 1 × 1 cm. Cancer invasion was confined to the submucosa (T1b). Toatl twenty mesenteric lymph nodes were examined, with carcinoma metastasis identified in 2 of 20 nodes (N1). All examined margins (gastric, duodenal distal, common bile duct, pancreatic) were negative for carcinoma. The transverse colon specimen was negative for carcinoma. Immunohistochemical analysis demonstrated strong positivity for CK7 (cytoplasmic), EGFR (cytoplasmic/membranous), and Ki-67 proliferation index of approximately 40% (Figure 2). All examined margins (gastric, duodenal distal, common bile duct, pancreatic) were negative for carcinoma.

### Postoperative complications and long-term adjunctive supportive care

Following pancreaticoduodenectomy, the patient developed absence of bowel sounds, recurrent vomiting, and a significant increase in bilioenteric drainage. Drain output averaged 580 mL/day (range 350–850 mL/day), with triglyceride level of 4.8 mmol/L and amylase of 42 U/L, consistent with grade C lymphatic leakage according to ISGPS criteria. Conservative management, including total parenteral nutrition, somatostatin analogs, and drainage, was maintained for 21 days. Concurrently, the patient developed delayed gastric emptying (ISGPS Grade B), still unable to tolerate solid food on postoperative day 10. After multidisciplinary team discussion, traditional Chinese medicine acupuncture was initiated to promote gastrointestinal function recovery. Acupuncture points selected included bilateral Zusanli (ST36), Shangjuxu (ST37), Taichong (LR3), Hegu (LI4), and Yinlingquan (SP9). Concurrently, comprehensive supportive treatment was administered, including parenteral nutrition (dextrose, amino acids, fat emulsion, vitamins, albumin, etc.), enteral nutritional support (enteral nutrition solution), and anti-infective therapy with antibiotics such as meropenem.

**Figure 3:**
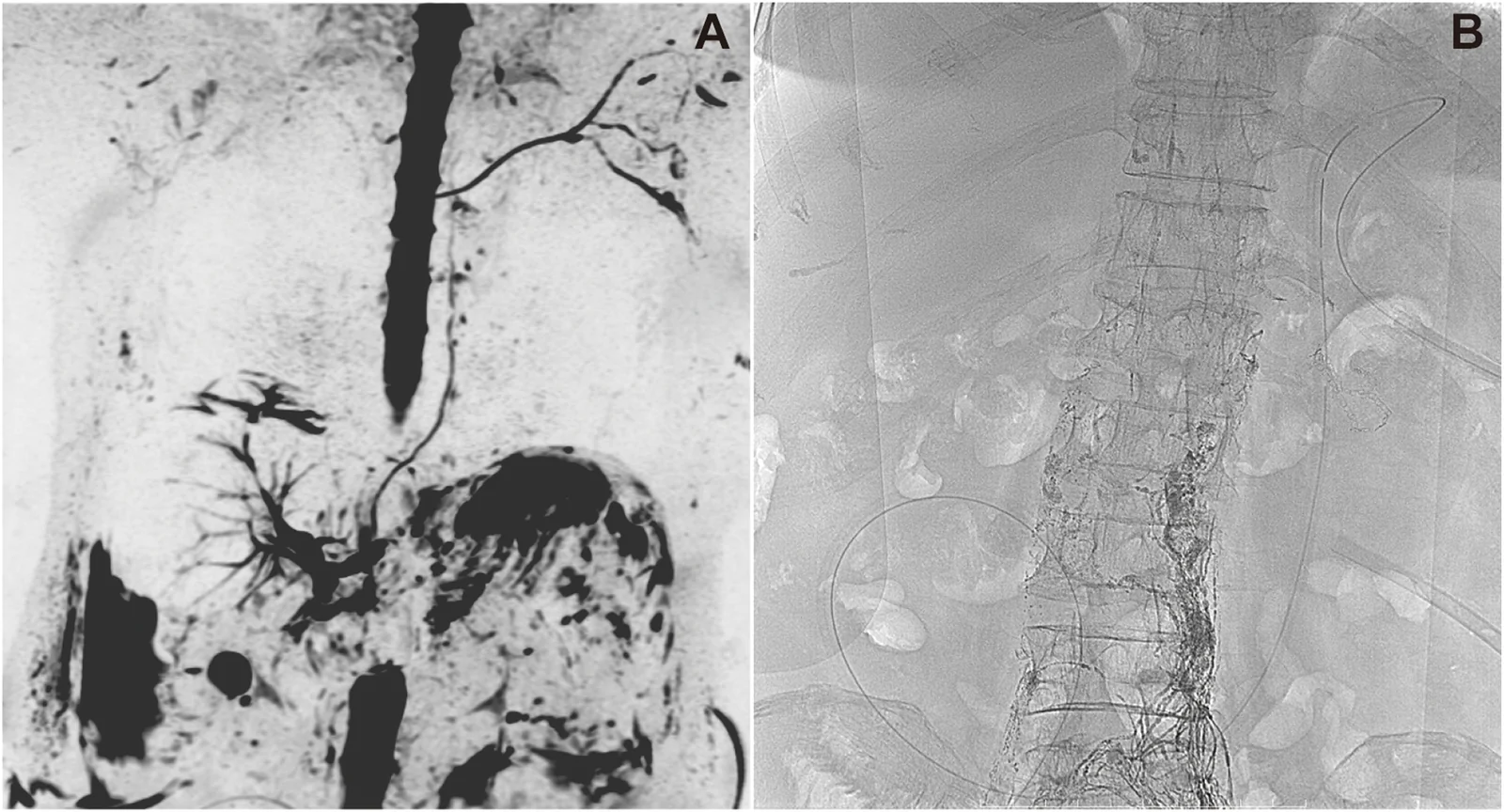
Lymphangiography and embolization for refractory lymph leak. **(A)** Digital subtraction angiography (DSA) showing abnormal lymphatic vessels. **(B)** Intra-procedural image during DSA lymphatic embolization.

As conservative management proved clinically ineffective, lymphatic vessel angiography and embolization were performed on postoperative day 22. The general procedure was as follows:Under color Doppler ultrasound guidance, a left inguinal lymph node was punctured. Iodinated oil was injected, and lymphangiography revealed satisfactory opacification of the left lumbar trunk, cisterna chyli, and thoracic duct, with contrast extravasation noted in the upper abdomen suggestive of a lymphatic fistula. Subsequently, the patient was repositioned prone, and the lymphatic fistula site was punctured. A mixture of tissue adhesive and iodinated oil was injected, and post-procedure angiography confirmed satisfactory embolization (Figure 3). Following embolization, drain output decreased dramatically from 580 mL/day to <50 mL/day within 72 h. The patient continued to receive comprehensive supportive care and gradually achieved complete transition to enteral nutrition by postoperative day 30. After three months of multidisciplinary management (from initial admission), the patient's condition was essentially stabilized. The chronological timeline of the entire treatment course is presented in Table 1.

**Table 1:** The chronological timeline of the entire treatment course of the presented case in this study.

Time point	Event
Day 0	Initial presentation to outpatient department
Day 1–3	Diagnostic workup (laboratory tests, MRI)
Day 4	Multidisciplinary discussion and patient counseling
Day 7	Stage I: Laparoscopic common bile duct exploration, stone extraction, cholecystectomy, intraoperative biopsy
Day 14–21	Postoperative cholangiography revealing complete obstruction
Day 22	Diagnosis of duodenal papillary adenocarcinoma confirmed
Day 25	Stage II: Radical pancreaticoduodenectomy with partial transverse colectomy
Day 1–14 post-PD	Postoperative period; development of lymphatic leakage etc.
Day 15–21 post-PD	Conservative management of lymphatic leakage (nutritional support, octreotide)
Day 22 post-PD	Lymphangiography and embolization
Day 25–30 post-PD	Progressive improvement; transition to enteral nutrition
Day 30–60 post-PD	Ongoing multidisciplinary rehabilitation (acupuncture, nutritional support)
Day 60–90 post-PD	Continued recovery; discharge planning
Day 90	Hospital discharge

## Discussion

Duodenal papillary adenocarcinoma is a malignant tumor originating from the glandular epithelium of the duodenal mucosa. With an incidence of less than 1 per 100,000, accounting for approximately 0.3%–0.5% of all gastrointestinal malignancies, it is considered a rare gastrointestinal cancer. The precise etiology of duodenal papillary adenocarcinoma remains unclear. Previous studies have indicated that advanced age (7), polyps (8), and biliary obstruction (9) are risk factors for this condition. In 2022, a research team described a case of duodenal papillary carcinoma coexisting with gallbladder carcinoma, detailing its diagnosis and successful treatment (10). Based on our knowledge and a review of domestic and international literature, there have been no prior reports on the clinical diagnosis and management of duodenal papillary carcinoma combined with cholelithiasis.

The non-specific symptoms associated with duodenal papillary adenocarcinoma lead to most patients being diagnosed at an advanced stage, resulting in a poor prognosis, high medical complexity, and elevated mortality. Particularly when combined with common bile duct stones, the clinical presentation of biliary obstruction is often overlooked (11). In clinical practice, when duodenal papillary adenocarcinoma coexists with other digestive tract diseases, a comprehensive and individualized diagnosis and treatment plan is essential (12). For early-stage duodenal papillary adenocarcinoma, radical pancreaticoduodenectomy is the primary treatment modality.

This report presents a case of a highly rare early-stage duodenal papillary adenocarcinoma discovered during the management of cholelithiasis in a primary care hospital. The patient initially presented with cholelithiasis and obstructive jaundice, with imaging suggesting a mass at the duodenal papilla. As the patient declined preoperative ERCP and biopsy, the treatment team adopted a staged strategy. First, laparoscopic common bile duct exploration with stone extraction and cholecystectomy were performed, with intraoperative biopsy providing a definitive diagnosis. Following pathological confirmation of duodenal papillary adenocarcinoma, a second-stage radical pancreaticoduodenectomy was performed. The staged surgical strategy was selected based on several clinical considerations. First, the patient presented with acute biliary obstruction requiring urgent decompression to prevent cholangitis and liver dysfunction. Second, while ERCP with biopsy represented the ideal diagnostic approach, the patient declined this invasive preoperative procedure after thorough counseling. Third, the primary care hospital setting limited immediate access to EUS-guided biopsy. The staged approach offered several advantages: (1) immediate relief of biliary obstruction; (2) obtaining definitive tissue diagnosis intraoperatively, addressing the patient's refusal of preoperative biopsy; (3) allowing recovery from acute biliary inflammation before undertaking major pancreaticoduodenectomy; and (4) providing time for comprehensive preoperative optimization, including nutritional support and management of comorbidities. However, this approach also carried disadvantages, including two separate surgical procedures with associated risks, potential tumor progression during the interval, and technical challenges from postoperative adhesions. The treating team carefully balanced these factors in a shared decision-making process with the patient and family. This strategy successfully addressed the acute biliary obstruction while creating the conditions for definitive diagnosis and curative surgery, reflecting a patient-centered and multidisciplinary approach.

Radical pancreaticoduodenectomy is a technically demanding major surgical procedure, often associated with a range of postoperative complications, including lymphatic leakage and gastroparesis, which are directly related to its extensive scope, significant trauma, and complex anatomy. In this case, the patient developed refractory lymphatic leakage postoperatively. Through multidisciplinary discussion, a dual approach was implemented: on one hand, traditional Chinese medicine acupuncture was utilized to aid gastrointestinal function recovery, alongside active nutritional support and anti-infective therapy (13); on the other hand, when conventional conservative management proved ineffective, the team promptly employed digital subtraction angiography percutaneous lymphangiography and targeted embolization. This technique precisely sealed the fistula, achieving satisfactory results. These outcomes highlight the unique value of modern interventional techniques in managing specific complex postoperative complications.

In summary, this case study describes the stepwise diagnosis of duodenal papillary carcinoma in a primary care hospital setting, initiated by the clinical symptoms of cholelithiasis. Through multidisciplinary discussion and thorough communication with the patient, an individualized treatment plan was formulated, involving staged surgery combined with adjuvant therapies. Through prolonged adjuvant care including traditional Chinese medicine, nutritional support, and meticulous nursing, satisfactory clinical outcomes were ultimately achieved. Previous studies have shown that radical pancreaticoduodenectomy combined with adjuvant therapy offers relatively good treatment efficacy for early-stage duodenal adenocarcinoma (14).

## Conclusion

Duodenal papillary adenocarcinoma is a rare gastrointestinal malignant tumor. Its early symptoms are often insidious, and it frequently coexists with common biliary diseases such as cholelithiasis, making it prone to missed or delayed diagnosis, with high incidence and mortality rates. This case report details the individualized clinical diagnosis and treatment of duodenal papillary adenocarcinoma combined with cholelithiasis in a primary care hospital. It highlights that for patients with duodenal papillary adenocarcinoma complicated by acute biliary obstruction due to conditions such as common bile duct stones, a staged surgical approach is a feasible individualized strategy. Furthermore, the management of complex complications following radical pancreaticoduodenectomy is crucial. This requires an integrated multidisciplinary diagnosis and treatment model in primary care hospitals, combining traditional supportive therapies with modern precision interventional techniques to improve patient prognosis. This case provides a significant reference for the comprehensive diagnosis and treatment of such rare and complex conditions.

## Data Availability

The original contributions presented in the study are included in the article/Supplementary Material, further inquiries can be directed to the corresponding authors.
